# Three-dimensional electron ptychography of organic–inorganic hybrid nanostructures

**DOI:** 10.1038/s41467-022-32548-x

**Published:** 2022-08-15

**Authors:** Zhiyuan Ding, Si Gao, Weina Fang, Chen Huang, Liqi Zhou, Xudong Pei, Xiaoguo Liu, Xiaoqing Pan, Chunhai Fan, Angus I. Kirkland, Peng Wang

**Affiliations:** 1 National Laboratory of Solid State Microstructures, Jiangsu Key Laboratory of Artificial Functional Materials, College of Engineering and Applied Sciences and Collaborative Innovation Center of Advanced Microstructures, Nanjing University, Nanjing, 210093 China; 2 Department of Materials, University of Oxford, Parks Road, Oxford, OX1 3PH UK; 3 College of Materials Science and Engineering, Nanjing Tech University, Nanjing, 210009 China; 4 Shanghai Key Laboratory of Green Chemistry and Chemical Processes, Department of Chemistry, School of Chemistry and Molecular Engineering, East China Normal University, Dongchuan Road 500, Shanghai, 200241 China; 5 The Rosalind Franklin Institute, Harwell Campus, Didcot, OX11 0FA UK; 6 School of Chemistry and Chemical Engineering, Frontiers Science Center for Transformative Molecules and National Center for Translational Medicine, Shanghai Jiao Tong University, Shanghai, 200240 China; 7 Department of Materials Science and Engineering, and Department of Physics and Astronomy, University of California, Irvine, 92697 CA USA; 8 Electron Physical Sciences Imaging Centre, Diamond Light Source Ltd., Harwell Science and Innovation Campus, Didcot, OX11 0DE UK; 9 Department of Physics, University of Warwick, Coventry, CV4 7AL UK

**Keywords:** Organic-inorganic nanostructures, DNA nanotechnology, Transmission electron microscopy

## Abstract

Three dimensional scaffolded DNA origami with inorganic nanoparticles has been used to create tailored multidimensional nanostructures. However, the image contrast of DNA is poorer than those of the heavy nanoparticles in conventional transmission electron microscopy at high defocus so that the biological and non-biological components in 3D scaffolds cannot be simultaneously resolved using tomography of samples in a native state. We demonstrate the use of electron ptychography to recover high contrast phase information from all components in a DNA origami scaffold without staining. We further quantitatively evaluate the enhancement of contrast in comparison with conventional transmission electron microscopy. In addition, We show that for ptychography post-reconstruction focusing simplifies the workflow and reduces electron dose and beam damage.

## Abstract (web summary)

The authors demonstrate electron ptychographic computed tomography by simultaneously recording high contrast data from both the organic- and inorganic components in a 3D DNA-origami framework hybrid nanostructure.

## Introduction

Structural DNA nanotechnology using templating techniques for material construction, was first proposed in the early 1980s by Seeman^1^ and has rapidly developed since 2006^2^. This development of DNA origami technology has shown great potential in a wide range of applications, including biomedicine^3–5^, biocomputing^6^, biochips and nano-circuits^7,8^, biomimicry^9^, masks for lithography^10,11^, and nano-mechanics^12^. All of these applications are based on the fact that a single DNA strand can be self-assembled into different two-dimensional (2D) and three-dimensional (3D) structures. In addition, spherical nanoparticles can be embedded in these structured DNA molecules as reported by Mirkin et al.^13^ and Alivisatos et al.^14^ and nanoparticle-capped DNA can be utilised as building blocks to build highly-ordered 3D lattice structures^15,16^, with a range of novel properties^17–19^. The physical properties of these are fundamentally determined by the relationship between the inorganic and biological components in the nano hybrid constructs which requires high-resolution 3D imaging of both the biological and non-biological components for characterisation.

Transmission electron microscopy (TEM), is a high resolution imaging technique, and is regarded as an essential tool for the characterisation of both biological and non-biological materials. Three dimensional imaging methods, such as single particle analysis (SPA)^20^ and electron tomography (ET)^21,22^, are widely used for 3D structural analysis of biological samples, and have recently been used in studies of DNA based structures^23,24^. SPA reconstruction, relies on computationally averaging thousands of images of identical particles (e.g., virus), and is more challenging to apply to heterogeneous samples with low symmetry. In contrast, ET, which is performed by recording a tilt series of projections of a single specimen, is more suitable for reconstructing the structure of non-identical biological samples such as 3D frameworks of DNA origami. Nevertheless, both of these approaches rely on the use of conventional phase contrast TEM images. It is well known that unstained biological samples, which are extremely radiation-sensitive and can be treated as pure phase objects. This offen results in images with low signal-to-noise ratio and low contrast, which can cause difficulties in the tomographic reconstruction. To enhance the contrast, samples of DNA origami frameworks in previous studies were prepared with staining^25,26^. However, the heavy metal ions used in the staining procedure can potentially damage the fine structure of native-state DNA^27^. An alternative to increase contrast at low spatial frequencies is to use high defocus values, but this leads to rapid oscillations in the phase contrast transfer function (CTF) at intermediate and high spatial frequencies if not corrected, for instance, by exit-wave restoration methods^28^. This poses a challenge to directly provide high-resolution 3D images of DNA frameworks, and to simultaneously visualise both the biological and non-biological components in their native state without staining.

Ptychography, as originally proposed by Hoppe^29^, is an alternative phase retrieval method related to coherent diffraction imaging^30,31^. The development of iterative algorithms^32–34^ has made ptychography capable of achieving superior spatial resolution compared to that obtained from conventional optics^31^, especially for extended non-crystalline samples^32^. Ptychography is now widely used in 2D imaging using both light^35^ and X-rays^30,32^ and in 3D imaging^36–40^. In TEM, it has also attracted considerable interest with a variety of potential applications in super-resolution imaging^41–45^, high-contrast light-element detection^46,47^, optical sectioning^46,48^, coupling to spectroscopic data acquisition^49^ and low-dose imaging^50–52^, using direct electron detectors^53,54^. Recent numerical simulations have shown that combining ptychography and electron tomography has the potential to reach atomic 3D reconstructions for inorganic materials^55^. Importantly, ptychography has a tunable and continuous wide-band information transfer, which can be adjusted using the probe-forming convergence angles which is important for biological applications. To transfer information from objects with larger length scales, the illumination can be further optimised to a smaller convergence angle^56^. This advantage has recently been demonstrated for organic matter (such as unstained virus particles), which were successfully reconstructed at a dose of 27 e^−^/Å^2^ in their native state^56^. We now propose that high-contrast 2D phase data reconstructed using ptychographic methods and recorded at different sample orientations can be aligned and used as an input to a tomographic reconstruction for unstained biological samples (such as DNA origami frameworks) with high resolution.

We demonstrate high-contrast 3D phase reconstructions to visualise unstained DNA origami with gold nanoparticles by combining electron ptychography and tomography. In comparison to conventional TEM, the enhancement of contrast was quantitatively evaluated under various electron dose conditions and accelerating voltages. Ptychography not only provides images with better contrast, but also simplifies the conventional tomographic workflow by reducing the electron dose and preventing radiation damage during tilting. Thus, electron ptychographic tomography (EPty-Tomo) developed as described here shows promise for revealing the relationship between 3D structures of hybrid nano-constructs fabricated with biomolecules and inorganic nanomaterials as used in aspects of nanotechnology.

## Results

### 3D ptychographic tomography (EPty-Tomo)

**Fig. 1:**
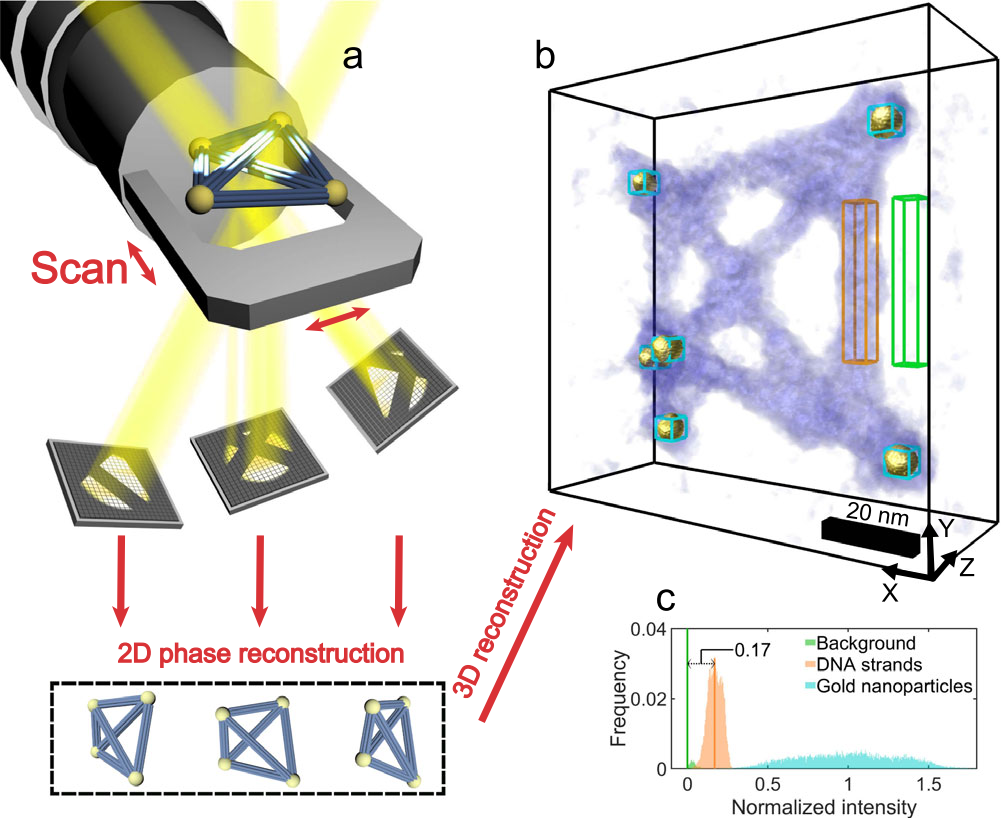
Schematic experimental setup and reconstruction of electron ptychographic tomography. **a** Schematic diagram of the experimental setup. **b** 3D rendering of the phase-contrast of a DNA origami framework reconstructed from a ptychographic tomographic dataset. **c** Frequency histogram of the background (green), DNA strands (orange) and gold nanoparticles (light blue) indicated by coloured boxes in (**b**).

The schematic experimental setup used is illustrated in Fig. 1a. Samples of unstained DNA origami with gold nanoparticles were suspended on an ultrathin carbon film at room temperature for TEM studies (see Supplementary Note 1: Sample Preparation). A tilt series of ptychography-based tomography was acquired using a + /− 70° double tilt holder (Fischione Model 2040) on a double aberration-corrected FEI Titan Cubed G2 60–300 (S)TEM operated at 60 kV accelerating voltage to reduce beam damage. A defocused electron probe with a diameter of approximately 120 nm was incident on the sample with a convergence angle of 1.5 mrad and a nominal defocus of −40 μm. The choice of a small convergence angle was to enhance low spatial frequency transfer^56^ with a large depth of field, which is an advantage when using thick specimens^57^. At each tilt angle, ptychographic datasets were acquired by illuminating the sample with a scanning probe, recording 10 × 10 far-field diffraction patterns (as shown in Supplementary Fig. 1) as a function of probe position (see Supplementary Note 2: Tilt-series Ptychographic Data Acquisition). This gave rise to a total ptychographic dataset for 23 tilt angles of approximately 39 GB. The 2D complex wavefunction of the sample at each tilt was recovered using the extended ptychographic iterative engine (ePIE)^33^ (see Supplementary Note 3: 2D Ptychographic Reconstruction). The phases of the tilt-series complex functions (see Supplementary Fig. 2) were used as inputs for sequential tomography reconstruction implemented using the GENeralized Fourier Iterative Reconstruction (GENFIRE) algorithm^58^ to calculate a final 3D reconstruction (see Supplementary Note 4: 3D Tomographic Reconstruction).

**Fig. 2:**
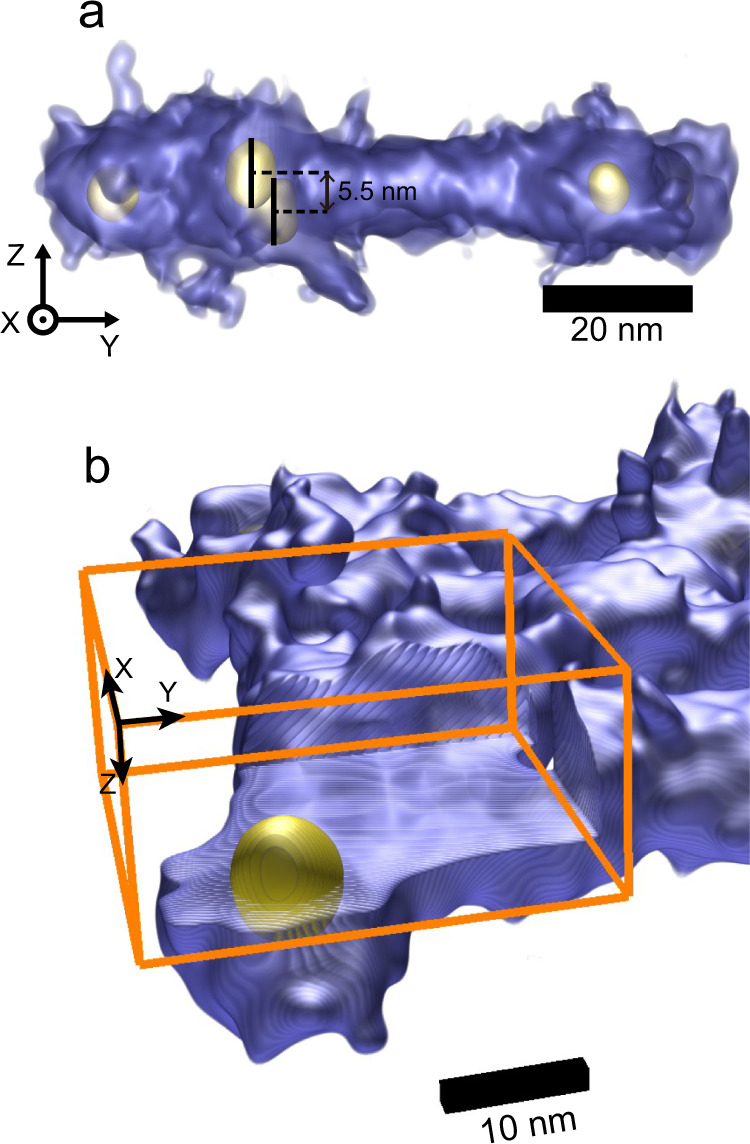
Sub-volume ptychographic tomography. **a** 3D rendering along the X-axis. **b** 3D rendering of a core-shell structure formed by a gold nanoparticle (yellow) and DNA strands (blue), which is hidden in the orange box.

The 3D phase reconstruction from ptychographic tomography is shown in Fig. 1b and Fig. 2. The 3D phase rendering of the unstained DNA origami structures with gold nanoparticles indicates that the DNA framework collapsed onto the carbon film due to the drop-cast sample preparation used. However, the side view and cross-section (Fig. 2a and Supplementary Fig. 3c) of the volume shows two adjacent gold nanoparticles spaced 5.5 nm apart along the z-axis. More importantly, the DNA strands together with the gold nanoparticles can both be resolved with high contrast in the 3D phase reconstruction. This is supported by the fact that the peaks of DNA strand and gold nanoparticles in the frequency histogram (Fig. 1c) are both separated from the background. A part of the reconstruction and its related video was also extracted, as shown in Fig. 2b and Supplementary Movie 1; Note that to reveal the gold particle core the signal of the DNA outer layer within the orange box is not rendered. An outer layer of signal surrounding the gold nanoparticles in the 3D phase reconstruction directly revealed the formation of core-shell structures in the scaffold. This outer layer of signal is attributed to a layer of DNA short strands formed from a spherical nucleic acid conjugate^59^. In contrast, ET, based on conventional defocused contrast TEM images (Supplementary Fig. 4), has difficulties in directly resolving the 3D structures of the biological and non-biological materials simultaneously. This is due to the low contrast of the biomolecules compared with that of gold nanoparticles, which will be discussed quantitatively in detail subsequently.

### Contrast quantification of DNA strands

**Fig. 3:**
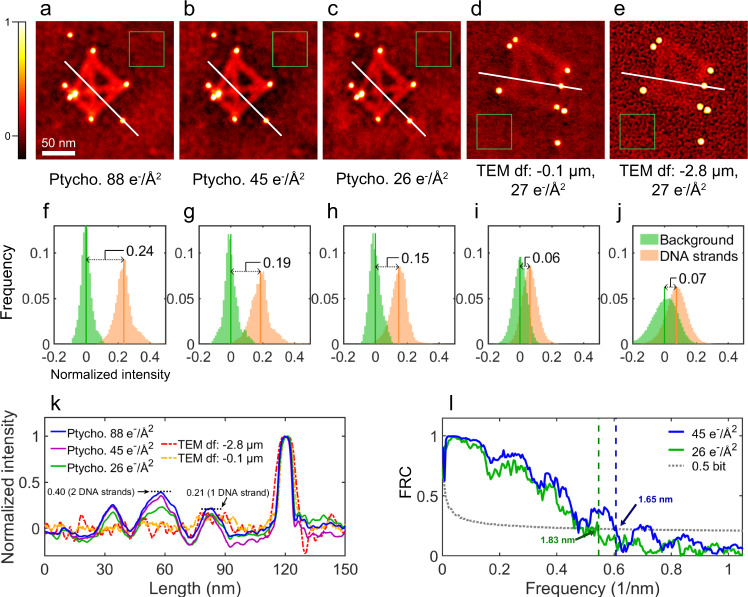
2D ptychographic phases and defocused TEM images. **a**–**c** Normalised 2D ptychographic phases with doses of 88, 45 and 26 e^−^/Å^2^, respectively. Reconstructed with independent randomly initialised phase guesses (as shown in Supplementary Fig. 9). **d**, **e** Normalised representative TEM images (from a set of typically 20 recorded) with doses of 27 e^−^/Å^2^ and defoci of −0.1 and −2.8  μm, respectively. **f**–**j** Frequency histograms of the background and DNA strands for **a**–**e**, respectively. **k** Line profiles extracted from the positions indicated by white lines in **a**–**e**. **l** Resolution estimates of the ptychographic reconstructions using Fourier ring correlation. Blue and green curves are calculated using two reconstructions at 45 and 26 e^−^/Å^2^, respectively.

In this study, EPty-Tomo exhibits two main advantages over conventional TEM based tomography. Firstly, reconstruction using ptychographic tomography shows an enhanced contrast from weakly scattering objects (unstained DNA strands), even though they are attached to strongly scattering material (gold nanoparticles). As a result, we could directly render the outer layer of the DNA short strands around the gold nanoparticles (Fig. 2b) which cannot be performed using conventional TEM tomography (Supplementary Fig. 4), unless the DNA strands are stained to boost contrast in the 2D TEM images that form the datasets used for tomographic reconstruction^60^. To quantify the contrast, the 3D phases were normalised with respect to the signals of the gold nanoparticles and the carbon film background (See Supplementary Note 5: Normalisation and Contrast Ratio). The signals of the carbon film background, DNA strands and gold nanoparticles were subsequently extracted from volumes with the equal number of the voxels as indicated with green, orange, and light blue frames in Fig. 1b and Supplementary Fig. 3, respectively. Finally, the normalised frequency histogram based on the extracted voxels was plotted as shown in Fig. 1c. The normalised phase of the DNA strands was localised within a narrow peak (orange) and was sufficiently high to be clearly distinguished from the carbon film background (green) by 0.17 (as a value of normalised intensity, this value is dimensionless. see Supplementary Note 6: Rendering and Histogram Analysis). This offset allows the contrast of the former to be directly separated from that of the latter in the 3D render. This result originates from the high-contrast 2D ptychographic phase reconstruction at each tilt (Supplementary Fig. 2), in which the contrast of DNA strands is higher than that of the conventional TEM images^56^. Figure 3a–c shows the normalised 2D ptychographic phase reconstructions at different electron doses of 88, 45 and 26 e^−^/Å^2^ varied by probe overlap ratios. As shown in the frequency histograms of the normalised data (Fig. 3f–h), the offset between the positions of the DNA and background peaks reduces from 0.24 to 0.15 with decreasing dose, confirming that the contrast in ptychographic reconstructions is dose-dependent^51^. As the data is normalised, the offset values can be considered as a contrast ratio (R), given by Eq. (1):

1 $${R}_{{DNA}/{gold}}=\frac{{P}_{{DNA}}-{P}_{{bkgd}}}{{P}_{{gold}}-{P}_{{bkgd}}}$$

where *R*_*DNA/gold*_ stands is the contrast ratio of the DNA strands and gold nanoparticles; *P*_*DNA*_ and *P*_*bkgd*_ is the intensity value (x-axis coordinate) at the peaks of the DNA strands and background in the frequency histograms, and *P*_*gold*_ is the mean value of the normalised intensity of a gold nanoparticle. Hence, larger *R*_*DNA/gold*_ values indicate higher visibility of the DNA strands with respect to the background and gold nanoparticles.

For comparison, a defocus-series of TEM images were also obtained over a range of defocus values from −0.1 μm to −8.9 μm (Supplementary Fig. 5a). The histograms of the original images (Supplementary Fig. 5b) shows that the contrast of the standalone DNA strands could only be increased by few tens of counts with increasing defoci. whereas, the peak positions of the gold nanoparticles at frequency histograms (Supplementary Fig. 5b) were increased by a few hundred in intensity. As a result, the *R* values remain low around 0.07 in the histograms (i.e. defoci of −0.1 and −2.8 μm, as shown in Fig. 3i, j, with data for other defoci shown in Supplementary Fig. 5e). Hence, the visibility of the DNA strands with respect to the gold nanoparticles is not improved with increasing defocus. In addition, larger defoci (−8.9 μm) leads to visible Fresnel fringes around the gold nanoparticles (shown in Supplementary Fig. 5a), which completely suppresses the contrast of the DNA short strands at the surface of gold nanoparticles. Thus, it is clearly challenging to resolve biological molecules around inorganic nanoparticles with high contrast and resolution using defocused TEM images. However, for the 2D ptychographic phases (Fig. 3a–c), the *R* value (0.24 – 0.15 in Fig. 3f–h) was two-fold higher than any defocused TEM data, so that DNA samples were imaged with higher contrast as shown in the line profiles across the DNA strands (Fig. 3k).

The ptychographic phase can reflect the relative thickness of the sample^61^. In this work, the normalised phase at the intersection of two DNA strands (0.40) is approximately twice that of a single DNA strand (0.21), as shown in Fig. 3k suggesting that the phase signal is quantitative for this sample^61,62^.

### Resolution of 3D reconstructions

The second advantage of EPty-Tomo is the capability to achieve a optimised resolution, the bandwidth of which can be controlled by changing the convergence semi-angle of the probe. To evaluate the 3D resolution, Fourier shell correlation (FSC)^63^, as conventionally used in tomography, was employed to evaluate the degree of correlation between two tomographic reconstructions at different spatial frequencies. The FSC was calculated using the full dataset (90 diffraction patterns at each projection) split into two independent datasets (45 diffraction patterns at each projection, as shown schematically in Supplementary Fig. 6) which were used for independent reconstructions, one of which is shown in Supplementary Fig. 7a. Details of the FSC analysis can be found in Supplementary Note 7: FSC and FRC and the FSC as a function of spatial frequency is shown in Supplementary Fig. 7b. Using this approach, the 3D resolution was estimated as 2.08 nm for a 0.5-bit criterion. It is established that the resolution of 3D reconstruction is determined by both the number of tilt angles and the resolution of individual 2D projections^64^. The theoretical resolution for 2D ptychography (two-dimensional projections in this 3D reconstruction) can be expressed as *λ*/(*α* + *β*), where λ is the electron wavelength, α is the convergence angle and β is the collection angle^65^. Since the DNA strands used here were weakly scattering objects and almost transparent to the electron beam, the signal scattered outside of the bright field disc is negligible. Therefore, in this work only the signal within the bright field disc is used for the ptychographic reconstruction, such that the 2D theoretical resolution, evaluated as *λ*/(2*α*) was 1.62 nm, in agreement with the result of experimental 2D phase-contrast resolution (Fig. 3i) achieved. The resolution of conventional phase contrast TEM imaging was defined by the first zero crossing of the contrast transfer function. For the 60 kV TEM image at a dose of 27 e^−^/Å^2^ (Fig. 3e) with the best *R* value (0.07 in Fig. 3j) obtained with a defocus of −2.8 μm without using an objective aperture, the resolution was estimated as 3.69 nm, which is worse than that (1.83 nm in Fig. 3l) of the ptychographic phase (Fig. 3c) under the similar dose condition of 26 e^−^/Å^2^ with almost a two-fold increase in the *R* value (0.19 in Fig. 3h).

### Contrast enhancement at low voltage

**Fig. 4:**
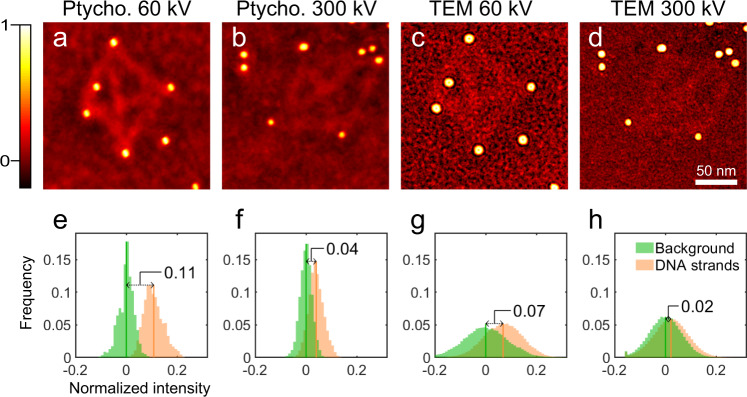
2D ptychographic phases and TEM images at various accelerating voltages. **a**, **b** Normalised ptychographic phases at accelerating voltages of 60 and 300 kV, respectively. **c**, **d** Normalised representative TEM images (from a set of typically 20 recorded) at accelerating voltages of 60 and 300 kV, respectively. **e**–**h** Frequency histograms of the images **a**–**d**, respectively.

As we have observed, both contrast and resolution were both strongly influenced by the accelerating voltage and the electron dose. Figure 4a–d shows comparisons between the normalised 2D ptychographic phases and TEM images taken at 60 kV and 300 kV accelerating voltages. From the same sample, the data at 60 kV showed a value of *R* approximately three times higher than that at 300 kV for both ptychography and TEM (Fig. 4e–h) consistent with increased contrast from weakly scattering objects at lower voltages^66^. Importantly *R* for the ptychographic reconstruction increased from 0.04 to 0.11, which was sufficient to distinguish the frequency peaks of the DNA strands from backgrounds at 60 kV, whereas for TEM data, it increased from 0.02 to 0.07, which did not meet the requirement for separation.

### Dose-dependent reconstructions

To study the dose-dependent ptychographic contrast of DNA, we reduced the total electron dose by reducing the overlap ratios (see Supplementary Note 8: Dose Reduction). As illustrated in Fig. 3a–c, the doses were 88 e^−^/Å^2^, 45 e^−^/Å^2^ and 26 e^−^/Å^2^ for each tilt angle, for overlap ratios of 83%, 76 and 66%. The resolution of 2D ptychographic reconstruction calculated by Fourier ring correlation (FRC) is shown in Fig. 3l (see details in Supplementary Note 7: FSC and FRC). Resolutions of 1.65 nm and 1.83 nm are calculated from two independent reconstructions with a dose of 45 e^−^/Å^2^ and 26 e^−^/Å^2^, respectively (Fig. 3l). Similarly, the contrast ratio *R* of the DNA strands was reduced from 0.24 (88 e^−^/Å^2^) to 0.19 (45 e^−^/Å^2^) and 0.15 (26 e^−^/Å^2^ as shown in Fig. 3f–h). By comparison, a series of TEM images with different doses (to over 250 e^−^/Å^2^) were also collected (Supplementary Fig. 8a). Using the same normalisation the contrast ratio (*R*) was maintained at around 0.065, when the dose was greater than 60 e^−^/Å^2^ (Supplementary Fig. 8c), suggesting that simply increasing the dose above this limit does not enhance the contrast of the DNA against the background of the amorphous carbon film in TEM imaging.

For 3D reconstruction, the total dose of the tilt series was reduced from 2024 e^−^/Å^2^ (Fig. 1b) to 1035 e^−^/Å^2^ (Supplementary Fig. 7a) and to 598 e^−^/Å^2^ (Supplementary Fig. 7d) using the dose-reduced 2D ptychographic phases (as shown in Fig. 3a–c). With reduced total dose (for details see Supplementary Note 8: Dose Reduction), the 3D structure of the DNA origami was still successfully reconstructed by ptychographic tomography. In a histogram analysis (shown in Supplementary Fig. 7c–f), the peaks were still separated although broadened. The 3D resolution calculated from a FSC was lowered from 2.08 nm at a dose of 1035 e^−^/Å^2^ (Supplementary Fig. 7c) to 3.74 nm at a dose of 598 e^−^/Å^2^ (Supplementary Fig. 7f) corresponding to the resolution degradation in the 2D phases (Fig. 3l). To further improve the quality of the reconstructed 2D phase data and at reduced dose, the use of a newly developed ultrafast detector^53,54,67–69^ shows promise. and recording data under cryogenic conditions will further limit radiation damage, as has been demonstrated by by Zhou et al.^56^.

## Discussion

To develop structural DNA nanotechnology, 3D TEM imaging is critical for precisely designing and characterising the programable inter-particle linkers, particularly at low electron dose. Generally, the 3D reconstruction methods that have been applied to DNA origami are either conventional tomography or SPA^70^ as summarised in Table 1. SPA is based on the assumption that the samples are homogeneous with many identical copies of the same structure. For example, the 3D reconstruction work by Shih et al.^71^ reported images of unstained DNA origami octahedral particles at 3.2 nm resolution, and assumed that many identical particles folded into the same structure^23,72^. However, DNA origami nanostructures are purposely designed and fabricated with multiple degrees of freedom and complex 2D/3D motion flexibility^73,74^ to diverse functionality and hence, SPA is unsuitable for reconstructing many different conformations. In contrast, TEM tomography is often chosen for 3D reconstruction of heterogeneous samples, as shown in Table 1. For example, Zhang et al.^24^ applied TEM tomography to study the structural dynamics of stained DNA-gold nanoparticle conjugates and achieved a 3D reconstruction with a resolution of 1.4 ~ 2.3 nm using a total dose of about 1500 ~ 2500 e^−^/Å^2^. However, the major problem of this approach is that the contrast of the DNA per se was not high enough to be distinguished from the support unless it was stained. This is consistent with our TEM observations of unstained DNA strands (Fig. 3d, e, Supplementary Fig. 4). However, taking advantage of the phase sensitivity of ptychography, we were able to achieve high contrast 3D reconstructions of unstained DNA origami at a resolution of 2.08 nm.

**Table 1:** Current status of 3D TEM techniques applied to DNA origami characterisation

	Ptychographic tomography (EPty-Tomo)	TEM tomography	Single particle analysis
Samples	DNA materials		
Sample requirement	Thickness estimated < 300 nm	Thickness < 300 nm^92^	Many identical instances
3D resolution	20.8 Å (this work)	14~23 Å^24^	23.5 Å^71^
		63 Å^26^	
Dose for 3D reconstruction	598~2024 e^−^/Å^2^ in total (this work)	1500~2500 e^−^/Å^2^ in total^24^ (Room temperature)	15 e^−^/Å^2^ per image^93^
		40~180 e^−^/Å^2 ^^26^ (Cryogenic)	

**Fig. 5:**
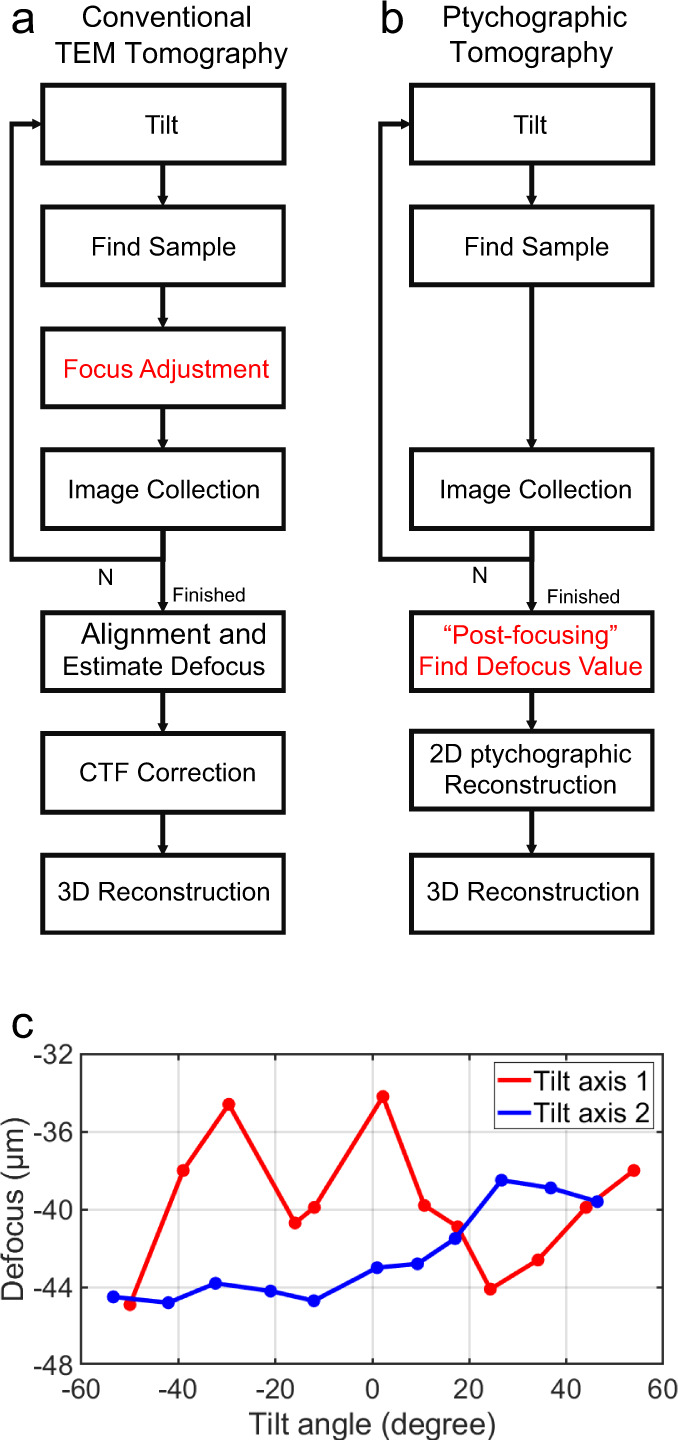
Workflows for tomography and post-acquisition correction of defocus. Workflows for tomography based on TEM images (**a**) and ptychography (**b**). **c** Defocus variations corrected by post-acquisition defocus adjustment as a function of tilt angle.

In general contrast and SNR in a tomogram of a biological sample are limited by the tolerance to total radiation dose during the entire acquisition of the entire tilt series. It is therefore worthwhile to compare the tomographic workflows that are used for conventional TEM with those for ptychography (shown respectively in Fig. 5a, b). For conventional TEM-based tomography, accurate defocus control is essential for high resolution image acquisition during tilting, as defoci need to be approximately constant for all tilt angles. However, in practice, the defocus value must be always tracked and adjusted before each image acquisition (Fig. 5a). This procedure can be time-consuming, but also adds electron dose that does not contribute to the final image dataset. In contrast, ptychography has the advantage of post-acquisition focusing^48^, and hence the defocus values can be compensated during the reconstruction after data collection (Fig. 5b). The defocus values for the current work were found to vary between −45 μm and −34 μm (Fig. 5c, Supplementary Table 1). Details of the post-acquisition focusing process are described in Supplementary Note 9: Post-focusing. As a result, defocus adjustment can be omitted during acquisition, saving exposure time and the dose budget.

As an alternative ptychographic retrival in STEM, phase-contrast methods including through focal series^28^ and tilt series^22,75^ have been used to increase resolution and improve image interpretation in TEM. Atomic resolution exitwave tomography has been theoretically demonstrated for an amorphous structure^76^. However, most of the experimental focus series exitwave reconstruction algorithms require small defocus changes relative to the dimensions of the sample^77^ in order to achieve high resolution^78^. To restore low spatial frequencies without compromising high spatial frequency transfer, the inclusion of a second focal series data set with a larger focal step has been suggested^28^. However, the large focal differences in the micrometre range required may lead to variations in magnification, image rotation, and possibly other distortions. In addition, the extent to which radiation damage may limit these alternative approaches remains to be explored experimentally, and the difficulties of image alignment at low dose may introduce further limitations.

In addition to computational methods, a physical phase plate such as Zernike^79–81^ and Volta^82,83^ that can be positioned at the back focal plane of objective lens to introduce a phase shift between the scattered and unscattered electron wave, leading to in-focus phase contrast without compromising low-resolution transfer. Tomography using phase plates is commonly used for the studies of biological objects in cryogenic TEM^81,84^. However, organic and inorganic hybrid structures such as the sample used here have not been studied. Furhermore these devices can suffer from signal attenuation at high frequencies, inconsistent fabrication, instability and a short usable life-span due to electrostatic charging^85,86^.

Recently, in STEM a pre-specimen phase plate was introduced in the probe-forming lens to acquire a 4D-STEM dataset with a focused probe, called MIDI-STEM^87^. This was further combined with non-iterative ptychography, giving enhancement of the contrast transfer at low spatial frequencies for 2D imaging^88^. Potentially this technique could further provide a 3D phase reconstruction when used in conjunction with tomography. Using MIDI-STEM also allows simultaneous annular dark field (ADF) imaging^89^, but it generally requires a fast detector. If raw data volumes in a dense representation are considered then there is also a substantial increase in data volume with field of view for this technique. However, we note that there have been reports of advanced data processing strategies to mitigate this^68,90^. In contrast, defocus-probe ptychography can be preformed with relatively simple detectors and requires less raw data volume for an object of the same size. Furthermore, removing the requirement to insert a condenser phase plate before each acquisition reduces the experimental complexity, particularly for low-dose experiments.

In summary, by combining electron ptychography and tomography, we have demonstrated 3D reconstruction of unstained DNA origami strands and gold nanoparticles with core-shell structures; a case of imaging biological and non-biological hybrid materials. In contrast to conventional TEM tomography, ptychography based tomography (EPty-Tomo) shows advantages of both high contrast and resolution at a low dose and can simultaneously visualise both weakly scattering materials (beam-sensitive biological materials) and strong scattering (inorganic, metal) materials. The post-focusing capability of ptychography simplifies the tomographic workflow, consequently reducing the total dose budget and radiation damage. With future developments of ultrafast detectors and improved cryogenic techniques, ptychographic tomography has great potential as an alternative to conventional electron cryo-tomography for high-resolution, large-volume visualisation of biological/non-biological hybrid structured materials. In turn this will assist the efficient development and refinement of fabrication processes and functional design.

## Methods

### Samples

DNA origami with a tetrahedral structure with gold nanoparticles inlayed was prepared using one-step self-assembly method. DNA origami was unstained and suspended on 50-mesh copper grid covered with an ultrathin carbon film in room temperature and air dried. Detailed information of sample preparation is described in Supplementary Note 1: Sample Preparation.

### Experimental measurements

Data for ptychographic tomography was acquired using a FEI Titan G2 Cubed with two aberration correctors at an accelerated voltage of 60 kV. A beam with a convergence angle of 1.5 mrad and defocus of −40 μm was formed giving a diameter of 120 nm at the sample. Diffraction patterns were recorded using Gatan Orius SC200 camera with 2048 × 2048 pixels for a dwell time of 0.2 s. At each tilt angle, the electron beam was scanned in a 10 × 10 array over the sample with a scanning step of 20 nm to fulfil the overlap ratio required by ptychography. The sample was tilted about two mutually perpendicular axes from −49.99 to 53.94 degree and from −53.42 to 46.40 degree, respectively. A Fischione Model 2040 tomographic holder was used which has a maximum tilt angle of +/− 70° degree. However, for higher tilting angles, the sample was blocked by the copper grid. The tilt angle increment was approximate 10 degree. Defocus values as a function of the tilt angles are listed in Supplementary Table 1 and typical diffraction patterns and 2D ptychographic phases are shown in Supplementary Fig. 1 and Supplementary Fig. 2.

### Ptychography and tomography reconstructions

The two-dimensional sample complex function at each tilt angle was reconstructed using the ePIE algorithm^33^. Detailed information of the ePIE algorithm used is described in Supplementary Note 3: 2D Ptychographic Reconstruction.

In the ptychographic reconstruction, amplitude of initial object guess is 1 (i.e. constant array), and initial phase is initialised with random low frequency information (from 0 to 0.035 nm^−1^). Initial probe function is calculated from the measured values of the convergence angle and defocus.

Diffraction patterns collected at each angle under different defocus conditions were processed by threshold and reconstructed using ePIE for 100 iterations with *α* = 0.5. Defocus values at each tilt angle are listed in Supplementary Table 1. The phases of the reconstructed complex functions (Supplementary Fig. 2) were then used as inputs for the subsequent tomographic reconstruction.

After ptychographic reconstructions, two datasets of the 2D ptychographic phase projections were generated from two tilt series as inputs for the tomographic reconstruction procedure. Tilt axes and image shifts were aligned manually using IMOD^91^ and tilt angles were subsequently calibrated (Supplementary Table 1). GENeralized Fourier Iterative Reconstruction (GENFIRE) algorithm^58^ was used for tomographyic reconstruction and the resultant 3D reconstructions is shown in Fig. 1b, Fig. 2, Supplementary Fig. 3a, Supplementary Fig. 7. Detailed information of tomographic reconstruction is described in Supplementary Note 4: 3D Tomographic Reconstruction.

### Reporting summary

Further information on research design is available in the Nature Research Reporting Summary linked to this article.

## Supplementary information

Supplementary information

Description of Additional Supplementary Files

Supplementary Movie 1

Reporting Summary

## Data Availability

The raw data (tilt-series diffraction datasets) in this study have been deposited in the Zenodo database (10.5281/zenodo.6819331).

## Code availability

Distributions of ePIE code are subject to an agreed licence agreement with Phase Focus Ltd. and the aurhors of this paper are not permitted to distribute the ePIE code used under the terms of that agreement. The IMOD code is available as open source under a General Public License (GPL) version v2.0 from the authors (https://bio3d.colorado.edu/imod/). The GENFIRE code is available as open source under a GNU General Public licence v3.0 (https://github.com/genfire-em/GENFIRE-Python).
