# Supplementary material for: Three-dimensional electron ptychography of organic–inorganic hybrid nanostructures
Source: Nat Commun. 2022 Aug 15;13:4787. doi: 10.1038/s41467-022-32548-x (PMC9378626; doi:10.1038/s41467-022-32548-x)
Supplement: Supplementary file 1 — Supplementary information [file 41467_2022_32548_MOESM1_ESM.pdf]

Supplementary Information

Three-dimensional Electron Ptychography of Organic-inorganic Hybrid Nanostructures

Supplementary Figures

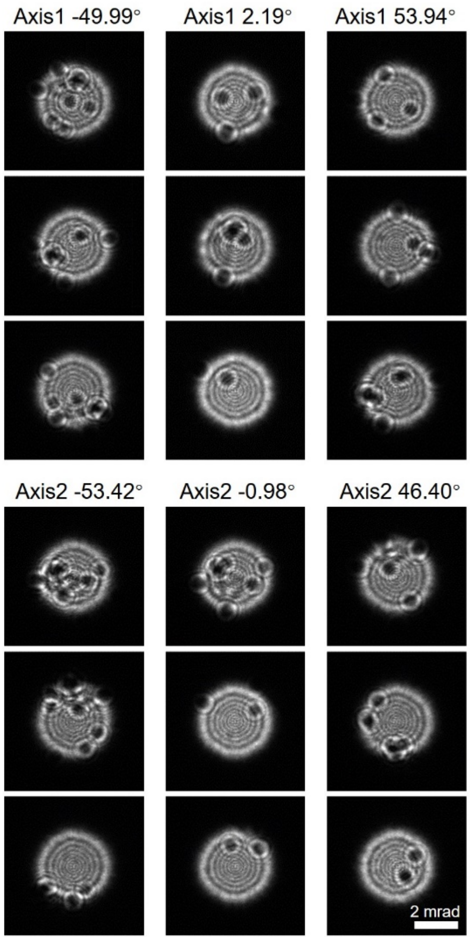

Supplementary Figure 1 | Typical diffraction patterns acquired at different tilt angles. Tilt axes and angles are indicated at the top of each column.

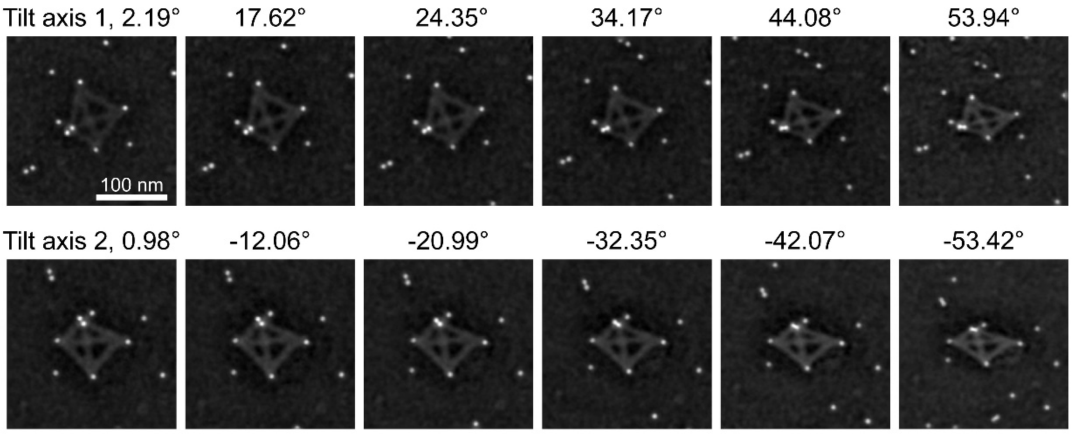

Supplementary Figure 2 | Phase of the complex wavefunctions reconstructed at various tilt angles. Tilt axis and angles are indicated at the top of each image. Scale bar is 100 nm.

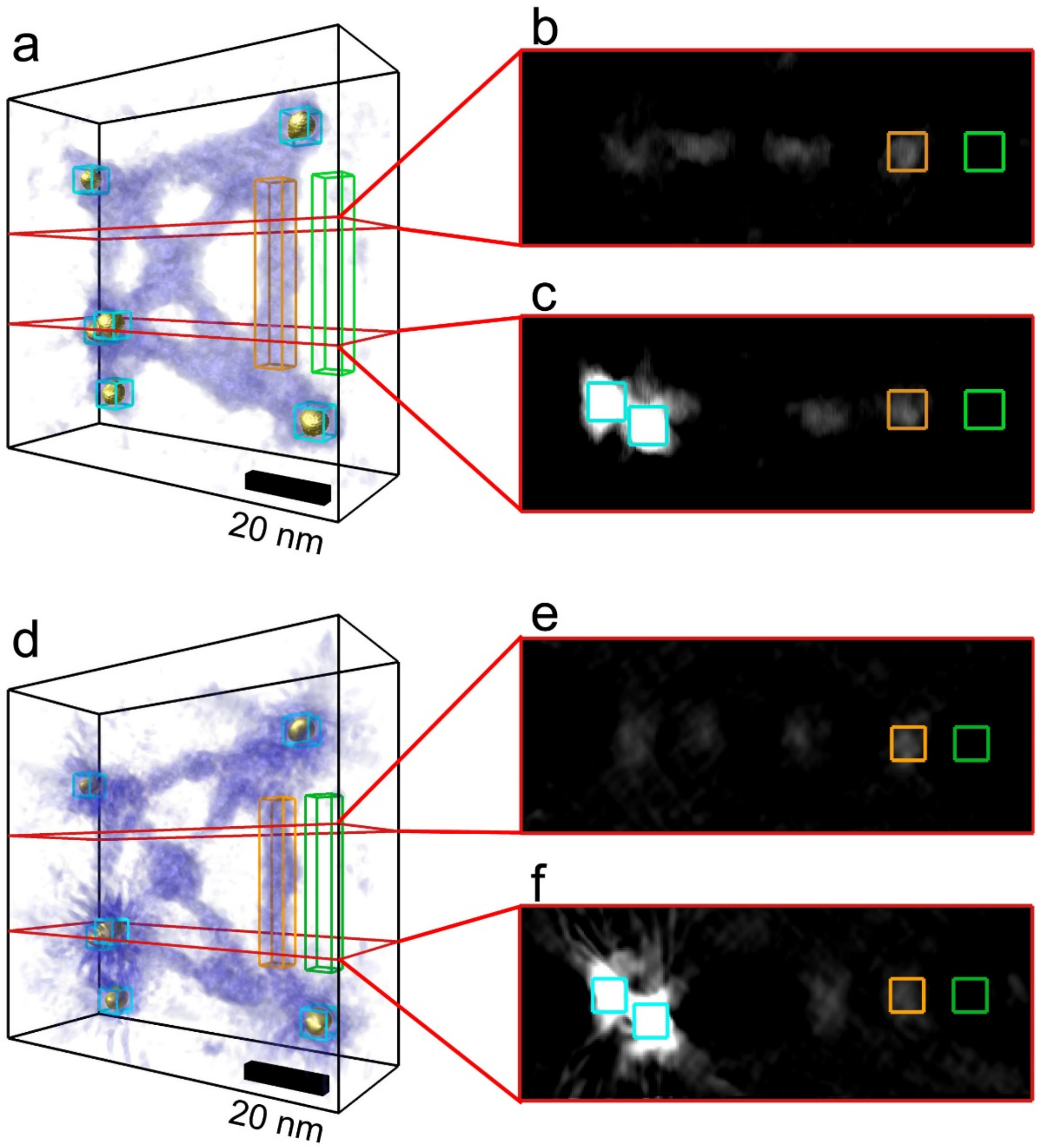

Supplementary Figure 3 | 3D reconstruction using GENFIRE and IMOD with cross sections. (a) 3D reconstruction obtained using GENFIRE. (b-c) Cross sections. (d-f) Reconstruction and cross sections obtained using IMOD.

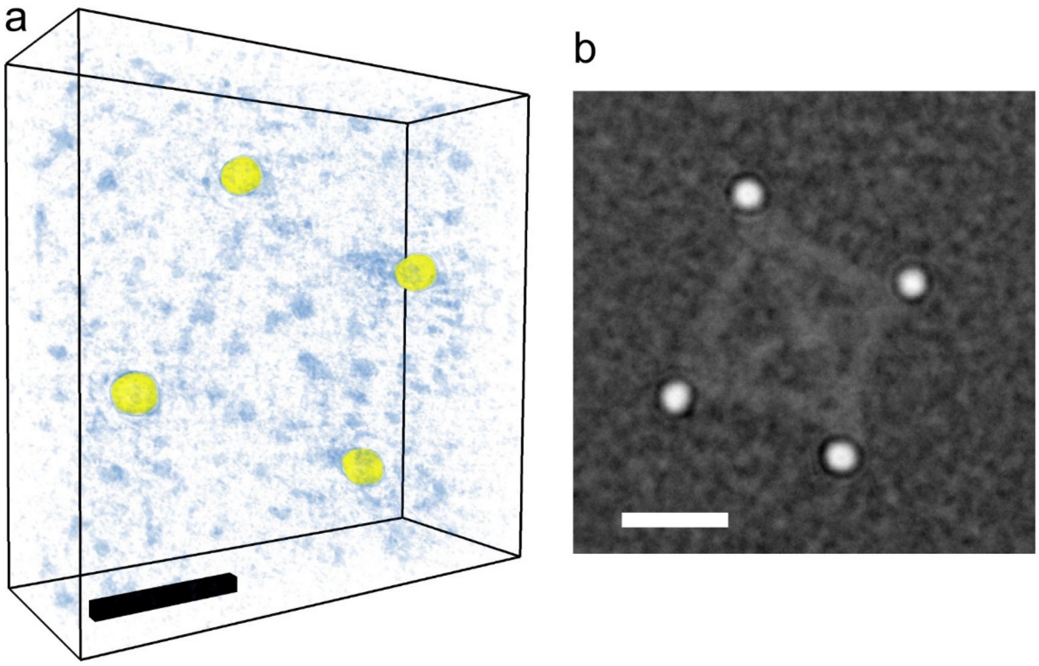

Supplementary Figure 4 | 3D tomographic reconstruction from defocused TEM data and one projection. (a) The 3D result reconstructed from 26 defocused TEM images along 2 tilt axes. The DNA origami can hardly be distinguished from the background. (b) TEM projection at a tilt angle of 0°. Scalebar is 50 nm. The TEM images were collected at 60 kV using the same camera that was used for the ptychography experiments. The pixel size of the projection is 0.724 nm. with a defocus value of -2 μm. 26 projections along 2 tilt axes were recorded (-59.44~64° for the first tilt axis and -66.03 ~ 58.20 for the second tilt axis). Total dose was 23 $e^-/Å^2$.

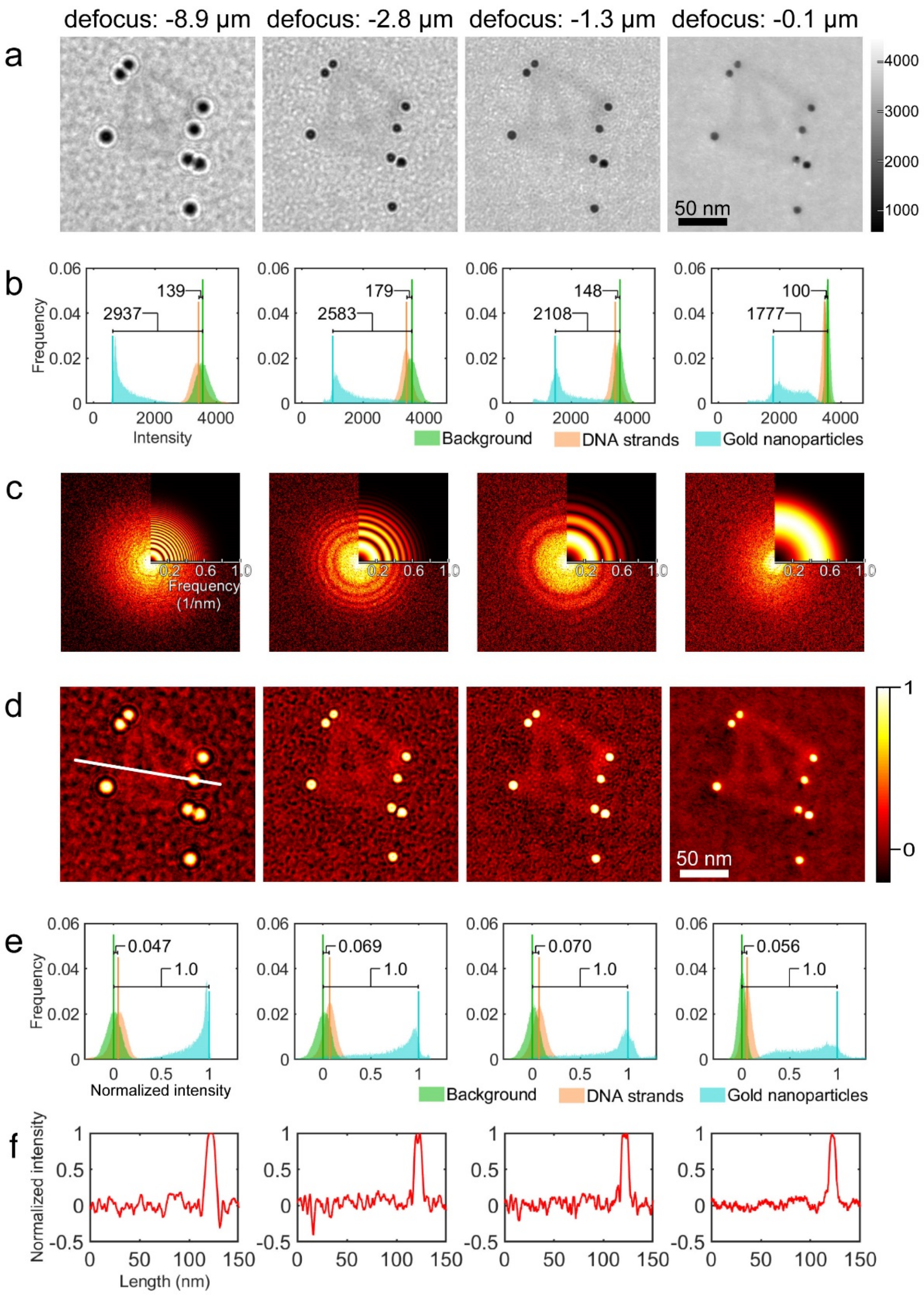

Supplementary Figure 5 | Defocus-dependent TEM images. (a) Series of representative original TEM images (without contrast reversal, from a set of typically 20 recorded) collected at 60kV at defoci from -0.1 to -8.9 µm. The electron dose is 27 $e^{-}/Å^{2}$. (b) Corresponding frequency histograms calculated from the images in (a). (c) Power spectra calculated from the images in (a) overlaid with their associated simulations. (d) Corresponding representative normalized TEM images (note that the contrast is reversed, from a set of typically 20 recorded). (e) Frequency histograms calculated from the images in (d). (f) line profiles taken at the positions of the white

line shown in (d).

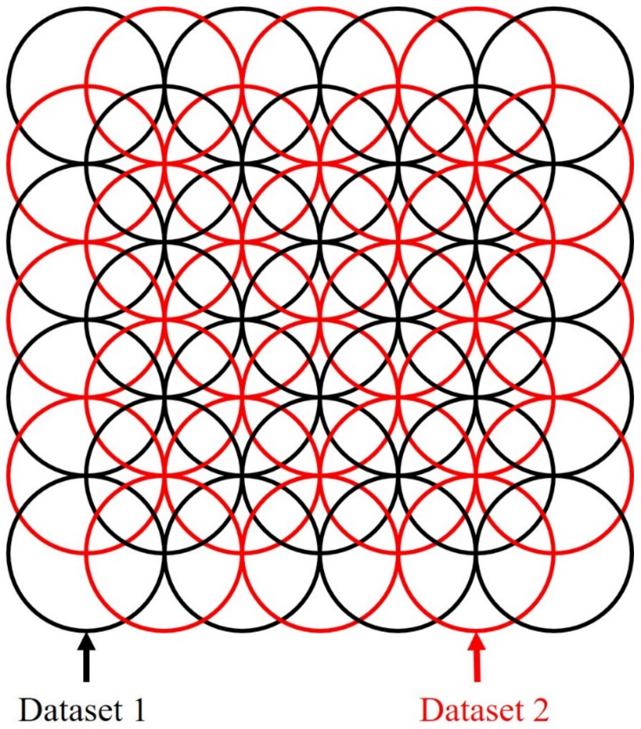

Supplementary Figure 6 | Schematic diagram showing a single ptychographic dataset split by selecting alternate measurements to estimate the resolution using FRC and FSC methods.

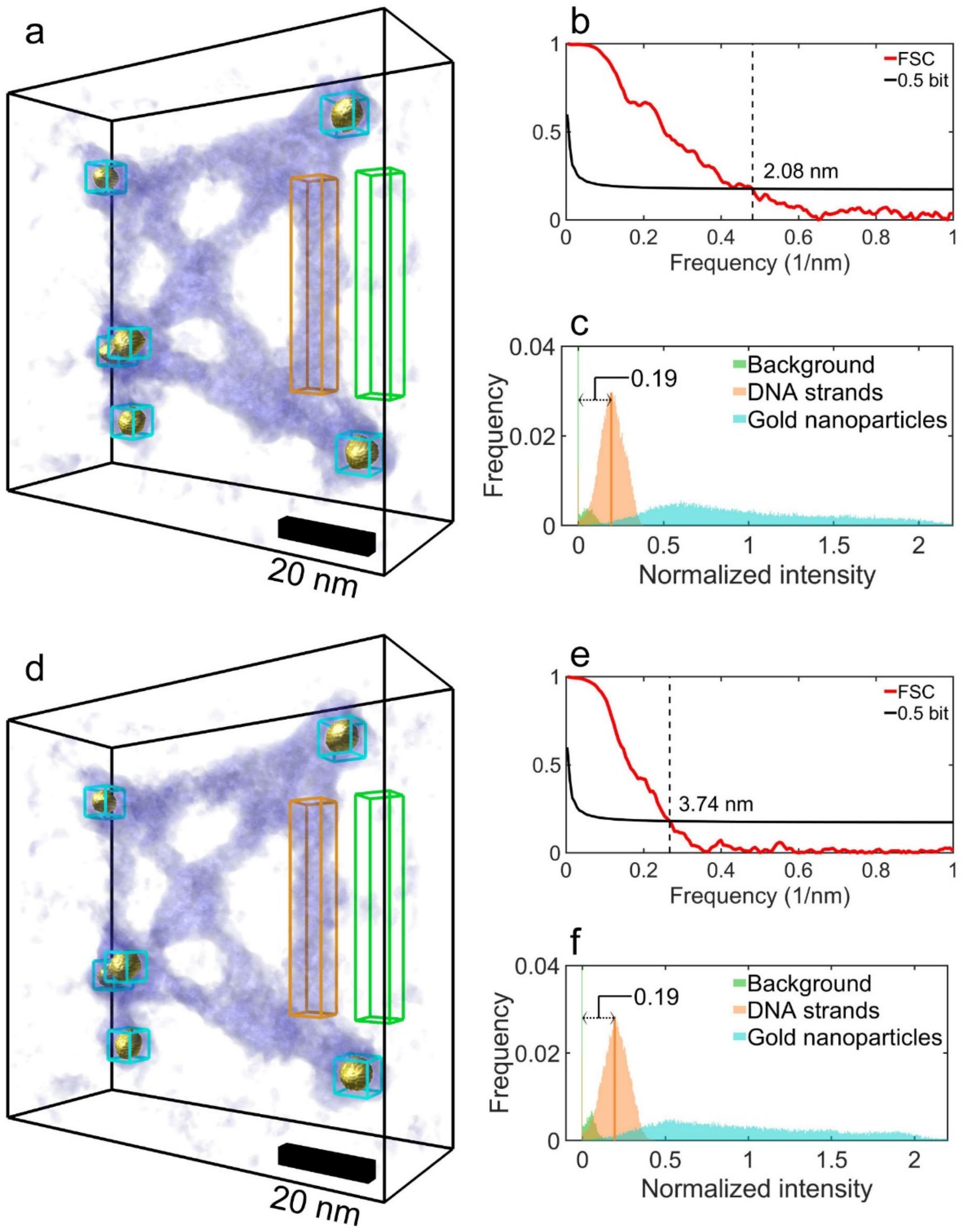

Supplementary Figure 7 | Dose-dependent 3D ptychographic phase calculated using GENFIRE. (a) 3D rendering of the 3D reconstruction, (b) FSC and (c) histogram for a total dose of 1035 $e^-/Å^2$. (d-f) Equivalents for a total dose of 598 $e^-/Å^2$.

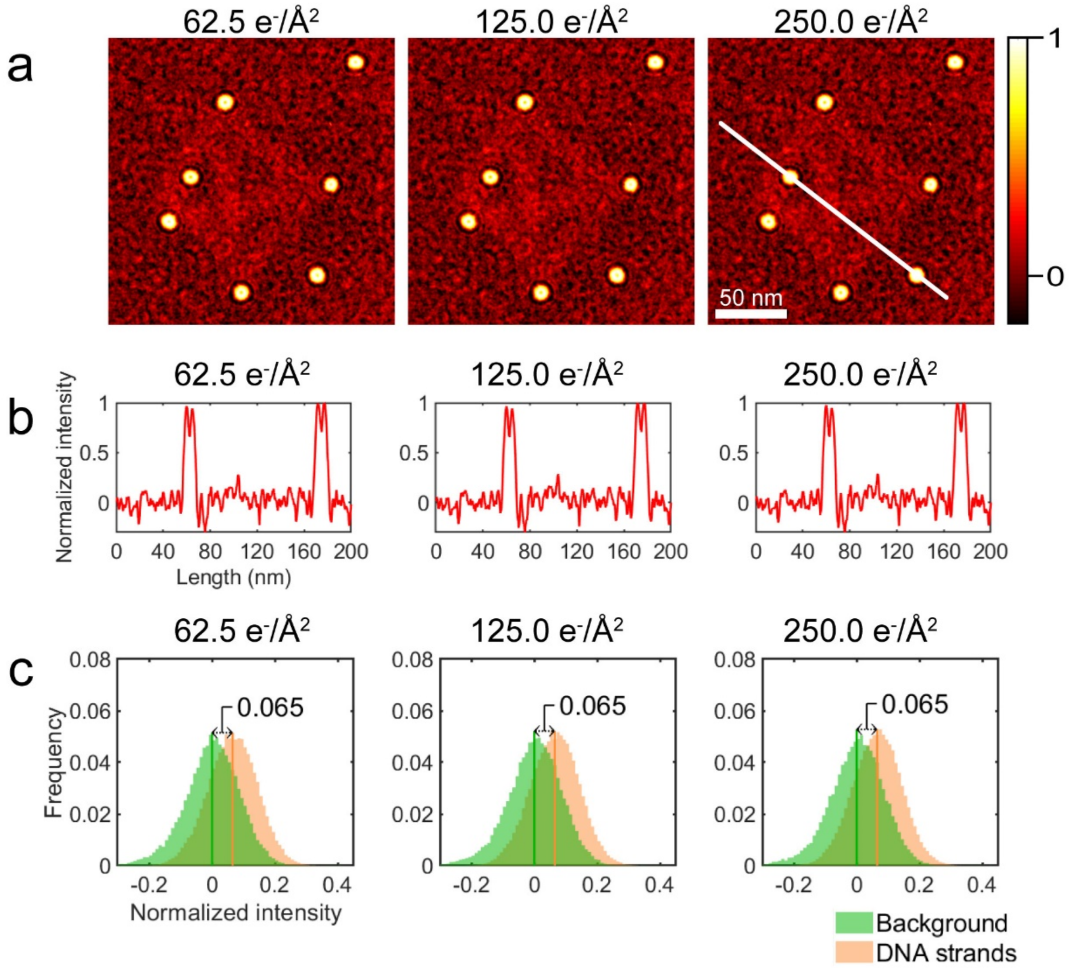

Supplementary Figure 8 | Dose-dependent TEM images. (a) Normalized representative TEM images (from a set of typically 20 recorded) with their contrast reversed for a defocus of -4 µm. (b) Line profiles extracted from the positions indicated with the white line in (a). (c) Corresponding frequency histograms calculated from the images in (a).

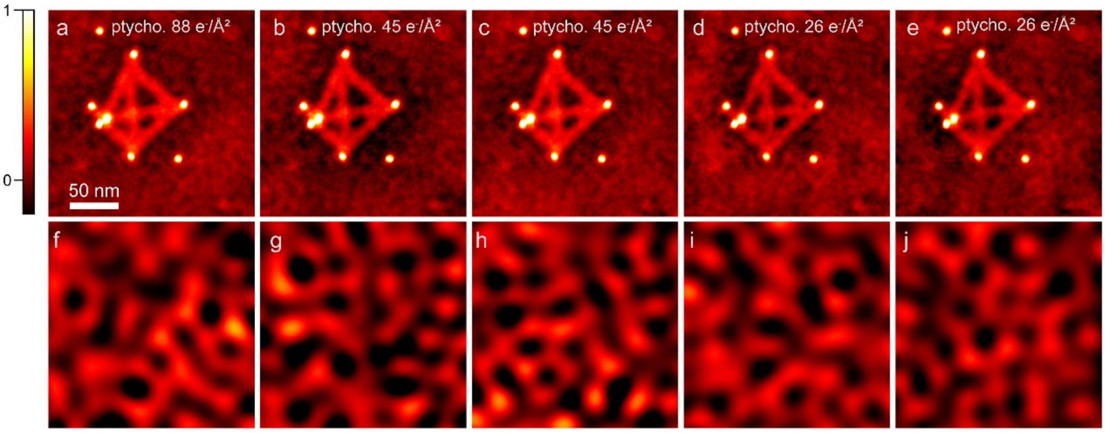

Supplementary Figure 9 | 2D ptychography phase reconstructions with randomly initialized phase maps. 2D ptychography phase reconstructions at doses of 88 e$^-$/Å^2 (a), 45 e$^-$/Å^2 (b & c), and 26 e$^-$/Å^2 (d & e). (f-j) The corresponding initial guesses of phase used in the reconstructions for (a-e).

Supplementary Table 1 | Post-acquisition defocus adjustments as a function of tilt angles.

Axis 1		Axis 2	
Calibrated tilt angle (degree)	Adjusted defocus (μm)	Calibrated tilt angle (degree)	Adjusted defocus (μm)
-49.99	-44.9	-53.42	-44.5
-39.00	-38.0	-42.07	-44.8
-29.61	-34.6	-32.35	-43.8
-15.94	-40.7	-20.99	-44.2
-11.96	-39.9	-12.06	-44.7
2.19	-34.2	0.98	-43.0
10.74	-39.8	9.32	-42.8
17.62	-40.9	17.10	-41.5
24.35	-44.1	26.65	-38.5
34.17	-42.6	36.80	-38.9
44.08	-39.9	46.40	-39.6
53.94	-38.0		

Supplementary Note 1: Sample Preparation

DNA origami with a tetrahedral structure was prepared using one-step self-assembly method proposed by Linuma *et al.*[1]. Each DNA origami strand contains 16 parallel double-helices packed on a honeycomb lattice, with a length of *ca.* 100 nm and diameters of 9 to 12 nm. The synthetic method to inlay gold particles in these samples was descripted by Cutler *et al.*[2]. DNA origami was unstained and suspended on 50-mesh copper grid covered with an ultrathin carbon film in room temperature and air dried.

Supplementary Note 2: Tilt-series Ptychographic Data Acquisition

Data for ptychographic tomography was acquired using a FEI Titan G2 Cubed with two aberration correctors at an accelerated voltage of 60 kV. A beam with a convergence angle of 1.5 mrad and defocus of -40 μm was formed giving a diameter of 120 nm at the sample. Diffraction patterns were recorded using Gatan Orius SC200 camera with 2048 × 2048 pixels for a dwell time of 0.2 second. At each tilt angle, the electron beam was scanned in a 10 × 10 array over the sample with a scanning step of 20 nm to fulfil the overlap ratio required by ptychography[3]. The sample was tilted about two mutually perpendicular axes from -49.99 to 53.94 degree and from -53.42 to 46.40 degree, respectively. A Fischione Model 2040 tomographic holder was used which has a maximum tilt angle of +/- 70° degree. However, for higher tilting angles, the sample was blocked by the copper grid. The tilt angle increment was approximate 10 degree. Defocus values as a function of the tilt angles are listed in Supplementary Table 1 and typical diffraction patterns and 2D ptychographic phases are shown in Supplementary Fig. 1 and Supplementary Fig. 2.

Supplementary Note 3: 2D Ptychographic Reconstruction

The two-dimensional sample complex function at each tilt angle was reconstructed using the ePIE algorithm[3]. The probe and object functions are denoted as $P_i(\vec{r})$ and $O_i(\vec{r})$, respectively, where i is iteration number. Hence, the exit wave $\psi_i(\vec{r})$ of the sample can be expressed as:

$$\psi_i(\vec{r}) = P_i(\vec{r}) \cdot O_i(\vec{r})$$

(Supplementary Equation 1)

where $\vec{r}$ is a real-space coordinate vector in the sample plane. The wave function $\Psi_i(\vec{k})$ in the diffraction plane is given by a Fourier transform of $\psi_i(\vec{r})$ as:

$$\Psi_i(\vec{k}) = \mathcal{F}\{\psi_i(\vec{r})\}$$

(Supplementary Equation 2)

The modulus of $\Psi_i(\vec{k})$ is now replaced by the corresponding experimental diffraction pattern $I(\vec{k})$ to yield $\Psi_i'(\vec{k})$ as:

$$\Psi_i'(\vec{k}) = \frac{\Psi_i(\vec{k})}{|\Psi_i(\vec{k})|}\sqrt{I(\vec{k})}$$

(Supplementary Equation 3)

Subsequently, an updated exit wave function $\psi_i'(\vec{r})$ is given by an inverse Fourier transform of $\Psi_i'(\vec{k})$:

$$\psi_i'(\vec{r}) = \mathcal{F}^{-1}\{\Psi_i'(\vec{k})\}$$

(Supplementary Equation 4)

Both updated object function $O_{i+1}(\vec{r})$ and probe function $P_{i+1}(\vec{r})$ are calculated from the following:

$$O_{i+1}(\vec{r}) = O_i(\vec{r}) + \alpha \times \frac{P_i^*(\vec{r})}{|P_i^*(\vec{r})|_{max}^2} \times [\psi_i'(\vec{r}) - \psi_i(\vec{r})]$$

(Supplementary Equation 5)

$$P_{i+1}(\vec{r}) = P_i(\vec{r}) + \alpha \times \frac{O_i^*(\vec{r})}{|O_i^*(\vec{r})|_{max}^2} \times [\psi_i'(\vec{r}) - \psi_i(\vec{r})]$$

(Supplementary Equation 6)

where α is usually set between 0 and 1.

For the next iteration, the updated probe function is moved to the next position in the data set and the newly updated object is used as the initial estimate. Amplitude of initial object guess is 1 (i.e. constant array), and initial phase is initialized with random low frequency information (from 0 to 0.035 nm^{-1}). Initial probe function is calculated from the measured values of the convergence angle and defocus.

Diffraction patterns collected at each angle under different defocus conditions were processed by threshold and reconstructed using ePIE for 100 iterations with $\alpha = 0.5$. Defocus values at each tilt angle are listed in Supplementary Table 1. The phases of the reconstructed complex functions (Supplementary Fig. 2) were then used as inputs for the subsequent tomographic reconstruction.

Supplementary Note 4: 3D Tomographic Reconstruction

Two datasets of the 2D ptychographic phase projections were generated from two tilt series as inputs for the tomographic reconstruction procedure. Tilt axes and image shifts were aligned manually using IMOD[4] and tilt angles were subsequently calibrated (Supplementary Table 1). For comparison, the traditional filtered back projection (FBP) algorithm was used in IMOD without using any filter and logarithm representation. The resultant 3D reconstruction (Supplementary Fig. 3d) shows that the large missing wedge of information introduces elongation and ghost tail artifacts in the gold particle tomograms (Supplementary Fig. 3f). For this reason the GENeralized Fourier Iterative Reconstruction (GENFIRE) algorithm[5] was used instead and the resultant 3D reconstructions is shown in Fig. 1b, Fig. 2, Supplementary Fig. 3a, Supplementary Fig. 7. In this case the effects of the missing-wedge artifact were substantially reduced (Supplementary Fig. 3c).

Supplementary Note 5: Normalization and Contrast Ratio

To compare the contrast of different imaging methods, both 2D ptychographic phases and TEM images were normalized using the following method. The TEM images were contrast reversed before the normalization to provide a direct comparison of the contrast

of DNA strands and gold nanoparticles. The normalized image (I_n) of an original image (I_o) can be calculated as:

$$I_n = \frac{I_o - V_b}{V_g - V_b}$$

(Supplementary Equation 7)

In which V_b and V_g are the average values of background (amorphous carbon film) and gold nanoparticle areas, respectively in the original image (I_o).

Similarly, for the 3D reconstructions (Fig. 1b and Supplementary Fig. 7), V_b and V_g were calculated from chosen sub-volumes and the normalization of the 3D reconstructions follows the calculation above.

The frequency histograms are calculated from the normalized images. The frequency histograms reflect the proportion of pixels (or voxels for the 3D reconstruction) with different normalized intensities in all pixels (voxels). In the normalization process used, the mean value of background was set to 0. Therefore, peaks due to the background and the average signal from the gold nanoparticles in the frequency histograms are located at around 0 and respectively.

To further quantify contrast of DNA strands, we define the contrast ratio of DNA strands to the gold nanoparticles as:

$$R_{DNA/gold} = \frac{P_{DNA} - P_{bkgd}}{P_{gold} - P_{bkgd}}$$

(Supplementary Equation 8)

Where, R is the contrast ratio while P represents the normalised intensity corresponding to the peak signal in a frequency histogram. P_{DNA}, P_{bkgd} and P_{gold} are the intensities of DNA strands, background, and gold nanoparticles respectively. Based on this normalization process, the value of $R_{DNA/gold}$ is equal to the normalized intensity of DNA strand signal peak in the frequency histogram.

Supplementary Note 6: Rendering and Histogram Analysis

The final 3D ptychographic tomographic reconstructions were rendered in Avizo using thresholds to extract the DNA strands and gold nanoparticles from the background, as shown in Fig. 1b, Fig. 2, Supplementary Fig. 3 and Supplementary Fig. 7. The sections in Supplementary Fig. 3 show that the DNA strands can be clearly distinguished from the background even without any *priori* knowledge.

To quantify the contrast, the 3D reconstruction was normalized by setting the average intensity of the background to zero and that of the gold nanoparticles to 1 (see Supplementary Note 5: Normalization and Contrast Ratio). The histograms shown in Fig. 1c and Supplementary Fig. 7c and f were then calculated by extracting the signals within the same number of voxels from the background, DNA strands and gold nanoparticles. Since the background of the 3D GENFIRE reconstruction is already normalized to 0 during the GENFIRE reconstruction, the histogram of the background (Fig. 1c) is a strong peak localized at zero.

For the 2D ptychographic phase data and TEM images in Fig. 3, Fig. 4, Supplementary Fig. 5 and Supplementary Fig. 8, the frequency histograms of the carbon film background and DNA strands were calculated from selected areas using the normalized data.

Supplementary Note 7: FSC and FRC

To evaluate the resolution of the 3D reconstructions, Fourier Shell Correlation (FSC)[6] was used, which measures the degree of correlation between two 3D volumes at different spatial frequencies. The full dataset of DNA origami and gold nanoparticles (90 diffraction patterns per tilt angle) was divided into two independent datasets (45 diffraction patterns per tilt angle) as shown in Supplementary Fig. 6. Two ptychographic tomography reconstructions were then carried out independently and the FSC was calculated from these two independent 3D reconstructions. The FSC curve of the 3D reconstruction shown in Supplementary Fig. 7b calculated from two reconstructions with a dose of 1035 $e^-/Å^2$ (45 diffraction patterns per tilt angle). The 0.5-bit criterion[6]

was applied to quantify the 3D resolution. The FSC curve in Supplementary Fig. 7e was calculated from 2 datasets with a dose of 598 $e^-/Å^2$ (25 diffraction patterns per tilt angle) and the 0.5-bit criterion was also used to quantify the resolution.

To evaluate the 2D resolution of a ptychographic reconstruction, Fourier Ring Correlation (FRC) [6,7] was used. The diffraction patterns were again divided into two datasets and the phase was reconstructed independently. The FRC was then calculated from independent 2D ptychographic phase reconstructions. The FRC curve (blue line in Fig. 3l) was calculated from two independent phase reconstructions with a dose of 45 $e^-/Å^2$ (45 diffraction patterns). The 0.5-bit criterion[6] was applied to quantify the resolution of this FRC curve. The FRC curve (green line in Fig. 3l) was also calculated from two independent phase reconstructions with a dose 26 $e^-/Å^2$ (25 diffraction patterns) and the same 0.5-bit criterion was used to quantify the resolution.

To evaluate the reconstruction at low spatial frequencies using FRC, two independent reconstructions with doses of 45 $e^-/Å^2$ (Supplementary Fig. 9 b & c) and 26 $e^-/Å^2$ (Supplementary Fig. 9 d & e) used different low spatial frequency perturbations as the initial guesses (Supplementary Fig. 9 g & h and i & j, respectively)[8]. The low spatial frequency perturbations from 0 to 0.035 nm^{-1} were introduced in the initial guess (see Supplementary Note 3: 2D Ptychographic Reconstruction). However, in the reconstructions, the dip of FRC curves (Fig. 3l) only appears within the range of 0 to 0.01258 nm^{-1}. At the frequencies higher than 0.01258 nm^{-1}, FRC values increase to 0.97 and 0.89 for 45 $e^-/Å^2$ and 26 $e^-/Å^2$, respectively (Fig. 3l). This indicates that information with frequencies higher than 0.01258 nm^{-1} can be reconstructed by ptychography algorithm in this experimental setting, which can be understood within the framework of contrast transfer functions for ptychography calculated in previous studies [9,10].

Supplementary Note 8: Dose Reduction

To reduce the electron dose of 3D ptychographic tomography from 2024 $e^-/Å^2$ (Fig. 1b, Supplementary Fig. 3a) to 1035 $e^-/Å^2$ (Supplementary Fig. 7a-c) and 598 $e^-/Å^2$ (Supplementary Fig. 7d-f), the same number of tilt projections used for the 3D reconstructions was maintained but the dose for each projection was decreased. For the

reconstruction at a dose of 1035 $e^-/Å^2$, the reconstructed phase data for each 2D projections used in the tomographic reconstruction has a dose of 45 $e^-/Å^2$. The diffraction patterns for each 2D projection were extracted from complete datasets following the method shown in Supplementary Fig. 6. For the reconstruction at a dose of 598 $e^-/Å^2$, the dose in each projection was reduced to 26 $e^-/Å^2$ by increasing the scanning step size from 20 nm to 40 nm.

The dose of the 2D phase reconstructions in Fig.3b and c (45 $e^-/Å^2$ and 26 $e^-/Å^2$) is reduced by the same method as for the 3D reconstruction at 1035 $e^-/Å^2$ and 598 $e^-/Å^2$ (Supplementary Fig. 7). Simultaneously, the overlap ratios were reduced from 83% (90 diffraction patterns) to 76% (45 diffraction patterns) and 66% (25 diffraction patterns).

Supplementary Note 9: Post-focusing Procedures

The probe defocus can be calibrated during the ptychographic reconstruction process. In this work, the nominal defocus value was set at -40 μm. However, due to positional inaccuracies of the sample holder along the z-axis, there is often an error of a few μm in the defocus value when the diffraction patterns are recorded. After data collection, by changing the initial probe in the ePIE algorithm (i.e. changing the defocus value as a fitted aberration), we calibrated the defocus values of datasets at each tilt angle, as shown in Supplementary Table 1 and Fig. 5c.

To achieve this the scan step (S_{true}), i.e. the distance between two probe positions, were recorded. The accurate defocus value for each tilt angle can then be calculated from the scan step and the position shifts as follows:

(1) Reconstruct a ptychographic phase for every diffraction pattern with an estimated defocus value ($df_{estimate}$).

(2) Calculate the position shifts ($S_{measure}$) of adjacent phases using clear feature points.

(3) Calibrate the defocus value as $df_{calibrate} = \frac{df_{estimate} \times s_{true}}{s_{measure}}$.

This calibration process of defocus value for each tilt angle is automated and is carried out before ptychographic reconstruction of the whole dataset. Other post-focusing methods such as inverse-multislice method[11] could be also used but are computationally more expensive.

Supplementary References:

1 Iinuma, R. *et al.* Polyhedra self-assembled from DNA tripods and characterized with 3D DNA-PAINT. *science* **344**, 65-69 (2014).

2 Cutler, J. I., Auyeung, E. & Mirkin, C. A. Spherical nucleic acids. *Journal of the American Chemical Society* **134**, 1376-1391 (2012).

3 Maiden, A. M. & Rodenburg, J. M. An improved ptychographical phase retrieval algorithm for diffractive imaging. *Ultramicroscopy* **109**, 1256-1262 (2009).

4 Kremer, J. R., Mastronarde, D. N. & McIntosh, J. R. Computer visualization of three-dimensional image data using IMOD. *Journal of structural biology* **116**, 71-76 (1996).

5 Pryor, A. *et al.* GENFIRE: A generalized Fourier iterative reconstruction algorithm for high-resolution 3D imaging. *Scientific reports* **7**, 1-12 (2017).

6 Van Heel, M. & Schatz, M. Fourier shell correlation threshold criteria. *Journal of structural biology* **151**, 250-262 (2005).

7 Saxton, W. O. & Baumeister, W. The correlation averaging of a regularly arranged bacterial cell envelope protein. *Journal of Microscopy* **127**, 127-138 (1982).

8 Allars, F. *et al.* Efficient large field of view electron phase imaging using near-field electron ptychography with a diffuser. *Ultramicroscopy*, 113257 (2021).

9 Zhou, L. *et al.* Low-dose phase retrieval of biological specimens using cryo-electron ptychography. *Nat Commun* **11**, 1-9 (2020).

10 O'Leary, C. M. *et al.* Contrast transfer and noise considerations in focused-probe electron ptychography. *Ultramicroscopy* **221** (2021).

11 Gao, S. *et al.* Electron ptychographic microscopy for three-dimensional imaging. *Nature Communications* **8** (2017).
