# Supplementary material for: Three-dimensional electron ptychography of organic–inorganic hybrid nanostructures
Source: Nat Commun. 2022 Aug 15;13:4787. doi: 10.1038/s41467-022-32548-x (PMC9378626; doi:10.1038/s41467-022-32548-x)
Supplement: Supplementary file 2 — Description of Additional Supplementary Files [file 41467_2022_32548_MOESM2_ESM.pdf]

Supplementary Movie 1: 3D reconstruction result of DNA origami and gold nanoparticles
